# Impact of Motivational Interviewing Education on General Practitioners’ and Trainees’ Learning and Diabetes Outcomes in Primary Care: Mixed Methods Study

**DOI:** 10.2196/75916

**Published:** 2025-09-16

**Authors:** Isaraporn Thepwongsa, Pat Nonjui, Radhakrishnan Muthukumar, Poompong Sripa

**Affiliations:** 1 Department of Community, Family and Occupational Medicine, Faculty of Medicine, Khon Kaen University, 123, Mittraparb Road, Khon Kaen, 40002, Thailand, 66 43363588; 2 Academic Affairs, Faculty of Medicine, Khon Kaen University, Khon Kaen, Thailand; 3 Inverkeithing Medical Group, Inverkeithing, United Kingdom

**Keywords:** motivational interviewing, general practitioners, general practitioner trainees, diabetes management, primary care, behavioral change, web-based learning, brief motivational interviewing

## Abstract

**Background:**

Effective diabetes management requires behavioral change support from primary care providers. However, general practitioners (GPs) often lack training in patient-centered communication methods such as motivational interviewing (MI), especially in time-constrained settings. While brief MI offers a practical alternative, evidence on its impact among GPs and patient outcomes remains limited.

**Objective:**

This study aimed to evaluate the effectiveness of a structured MI educational program for GPs and GP trainees on their MI knowledge and confidence, and its impact on clinical outcomes among patients with type 2 diabetes in primary care settings.

**Methods:**

A mixed methods study was conducted using a before-and-after two-group design with quantitative assessments of GPs’ knowledge and patients’ biomarkers, supplemented by qualitative interviews. The intervention group (n=35) received a 4-hour interactive MI workshop, optional web-based modules, and brief MI guides. The control group received standard care. A total of 149 and 167 patients with diabetes were included in the study and control groups, respectively.

**Results:**

A paired-sample *t* test was conducted to evaluate the impact of the MI course on the learners’ knowledge. There was a statistically significant difference in the knowledge test scores from Time 1 (mean 11.46, SD 3.48) to Time 2 (mean 15.04, SD 2.35), *t*_28_= –7.74; *P*<.001 (2-tailed). The mean increase in knowledge score was 3.57 (SD 2.44), with a 95% CI of 2.62 to 4.52, indicating a large and statistically significant effect. The eta-squared statistic indicated a large effect size (eta-squared=0.85). Patients in the intervention group had greater improvements in HbA_1c_ (mean difference= −0.50, 95% CI −0.91 to −0.09; *P*=.02) and diastolic blood pressure (mean difference= −5.96 mmHg, 95% CI −8.66 to −3.25; *P*<.001) compared to controls. Qualitative feedback highlighted the usefulness of brief MI, along with challenges in mastering advanced techniques and time constraints.

**Conclusions:**

The MI educational program improved GP trainees’ MI knowledge and patient outcomes. Brief MI appears feasible in primary care but requires ongoing support for skill development and implementation.

## Introduction

Diabetes is a chronic illness with a rising prevalence worldwide [1]. Over the past two decades, the global prevalence of diabetes has significantly increased. In 2021, it was estimated that 536.6 million people were living with diabetes [1]. This number is projected to rise to 642.7 million (11.3%) by 2030 and 783.2 million (12.2%) by 2045 [1]. Furthermore, 3 out of 4 adults with diabetes reside in low- and middle-income countries [1]. Approximately 240 million people are living with undiagnosed diabetes globally, with 90% of these individuals residing in low- and middle-income countries [1]. Diabetes and its complications, such as heart disease, stroke, and chronic kidney disease, are pressing public health challenges that are expected to worsen globally [2,3].

Patients with diabetes often face significant challenges in adopting and maintaining healthy lifestyle changes, which can hinder blood sugar control and increase the risk of complications. Research and systematic reviews have highlighted multiple barriers to sustained behavioral change, including low motivation, insufficient self-discipline, and limited adherence to recommended lifestyle practices [4-7]. These challenges are often compounded by a lack of personalized guidance and support for self-management from health care providers [8], as well as systemic constraints such as limited consultation time for effective behavioral counseling [9,10]. Many providers report difficulty engaging patients in meaningful discussions about lifestyle modification due to limited training in behavioral change techniques and patient-centered communication [9]. Addressing these issues requires strategies that not only convey information but also actively enhance patient motivation, strengthen self-efficacy, and support the development of sustainable health behaviors within routine care.

Motivational interviewing (MI), a counseling method widely recognized and developed by Miller and Rollnick [11], is a patient-centered approach [12]. MI focuses on the client’s perspective, allowing them to express their feelings and motivations for change [12]. It empowers clients to take ownership of their change process, thereby reducing defensiveness and resistance [12]. MI consists of 4 key processes: engaging, focusing, evoking, and planning [11]. The foundational skills of MI—open-ended questions, affirmations, reflections, and summaries—are used to improve engagement and facilitate change talk [11]. Focusing is the process of finding or discussing a common interest topic for behavioral change between the client and counselor [11]. In the evoking process, the counselor aligns with the patient’s talk (change vs sustain talk) to encourage change. The more the patient makes change talk, the closer she or he is to the change. Patients were guided, invited, and responded toward change talk, such as reflecting on past successes, envisioning future benefits, considering consequences, exploring extreme scenarios, identifying personal values, and addressing ambivalence [11]. During the planning process, the patient expresses more change talk and less sustain talk. In this process, potential obstacles to change and strategies to overcome them are explored [11]. This approach has been successfully applied to various lifestyle challenges and medical conditions [12,13]. Systematic reviews and meta-analyses have demonstrated efficacy of MI in addressing various health behaviors, including alcohol abuse, increasing physical activity, smoking cessation, pain management, and improving body weight and blood sugar control [14-17]. The application of MI varies across health care settings and among different clinicians. Reviews continue to support the positive impact of MI across diverse diseases, settings, and delivery modes, effectively delivered by various health care professionals [12,16,18].

General practitioners (GPs) or family physicians play a pivotal role in diabetes management [19]. Their involvement is essential for improving health outcomes, particularly by facilitating behavioral changes [20]. GPs are uniquely positioned to promote health and prevent disease through lifestyle counseling and guidance on managing risk factors [21,22]. However, previous studies have found that patient-centered, motivational techniques aimed at behavioral change are not routinely used during diabetes consultations [23,24].

Given their central role in supporting behavioral change, GPs are well-positioned to apply MI to promote lifestyle modification and improve outcomes in patients with type 2 diabetes. Previous studies have shown that MI delivered by health care professionals in primary care can improve diabetes management outcomes, including glycemic control, weight loss, and self-management [12,15,18,25,26]. When delivered specifically by GPs, a systematic review reported that MI may enhance patient outcomes; however, the effects are often modest and inconsistent, with variable results observed for HbA_1c_, cholesterol levels, and physical activity [17,27]. In addition, a significant research gap remains regarding MI’s impact on GPs themselves, particularly in terms of their knowledge, competencies, and barriers to implementation in routine diabetes care [17].

Despite its feasibility in daily general practice, the uptake of MI remains inconsistent. Common barriers include time constraints—especially in managing complex conditions like diabetes, which require detailed behavioral counseling—and the difficulty of balancing MI principles with competing clinical priorities during a single consultation [12,17]. Brief interventions have been suggested for use in routine diabetes care; however, these often lack the patient-centered techniques central to MI [20,28]. To address this, brief MI has been developed as an adapted form of MI for high-volume clinical settings [29]. It emphasizes core processes such as engaging and evoking within a condensed interaction, making it feasible within standard consultation times [29]. This adaptation enables GPs to facilitate patient-centered behavioral change without conducting a full-length MI session.

Brief MI may be a more practical approach for busy GPs in managing diabetes. Despite its potential, there is a notable lack of rigorous studies evaluating the effectiveness of brief MI in diabetes care. For instance, a study implemented brief MI but involved only 4 patients with diabetes and was delivered by providers who were not exclusively GPs [30]. Another study found that training brief MI for family medicine residents can enhance their counseling skills and improve patient self-management, as evidenced by the increased use of MI-adherent approaches and improved clinical outcomes [31]. Additional challenges to implementing brief MI include the complexity of the technique, which demands that GPs develop advanced clinical skills to apply MI effectively, and the lack of ongoing support systems to sustain and enhance these skills over time [17]. Addressing these barriers is critical for optimizing the feasibility and impact of brief MI in primary care settings.

Continuing medical education (CME) or the broader concept of continuing professional development (CPD) is widely used to maintain the quality of GPs’ clinical practice [32] and for GP diabetes education [33]. Multifaceted educational approaches, such as interactive workshops, small-group discussions, outreach visits, audits and feedback, and reminders, have been shown to effectively improve physician practices and clinical outcomes [34,35]. In contrast, traditional CME methods, such as lectures and educational material distribution, have a limited impact on behavioral change [34,35].

Web-based educational methods are increasingly popular due to their flexibility, convenience, and reduced travel costs, offering similar effectiveness to traditional methods [36,37]. While web-based CME methods may not replace traditional approaches, they can serve as complementary tools to enhance learning outcomes [36,38]. Previous studies have examined the effectiveness of web-based MI training across diverse diseases, settings, and health care professional groups [29,39-43]. These studies have shown the potential of web-based MI training to improve learners’ knowledge, skills [39,40], and confidence [29,42]. However, evidence on its impact on clinical practice or patient outcomes is scarce [39]. In addition, the role of web-based educational methods in supporting MI skill development for GPs managing diabetes has not been rigorously tested.

This study addresses existing gaps by implementing a comprehensive educational program for GPs, combining an interactive workshop, structured web-based learning, and practical guides for brief MI. The primary research question was whether this multifaceted MI educational approach could improve GPs’ learning and patient clinical outcomes. Therefore, this study aimed to evaluate the effectiveness of a structured educational program on MI for GPs and GP trainees in primary care settings. Specifically, it assessed whether a combination of interactive workshop training, web-based modules, and practical brief MI guides could improve participants’ MI-related knowledge and confidence, and whether this translated into improved clinical outcomes among patients with type 2 diabetes in pilot communities, and exploring GPs’ experiences, perceptions of the MI training, and their implementation of MI in routine consultations.

## Methods

### Study Components and Research Design

To address the overarching aim of evaluating the effectiveness and feasibility of MI training in primary care, this study used a mixed methods design composed of three components: (1) Evaluation of GP learning outcomes: A before-and-after study assessed changes in GPs’ and GP trainees’ knowledge and confidence related to MI through pre- and posttests; (2) Assessment of patient clinical outcomes: A quasi-experimental, nonrandomized 2-group design compared biomarker changes in patients with diabetes from intervention (MI-trained) and control (routine care) sites; and (3) Qualitative exploration of GP experiences: A descriptive qualitative study using semistructured interviews explored GPs’ experiences in applying MI in routine consultations after training and their experiences in the MI education. GP interviewees were selected based on their confidence in applying MI in routine consultation after their completion of the MI courses. This integrated structure is justified by the implementation nature of the study, aiming to evaluate not only educational effects but also practical application and outcome relevance. The integration is intentional and appropriate for a pragmatic, real-world educational intervention. Improving GP knowledge without understanding its translation into patient care would offer limited insights. Conversely, changes in patient biomarkers without a provider perspective could misattribute causality. Including qualitative data allowed us to contextualize the quantitative results, explain variability in outcomes, and identify barriers and facilitators to implementation.

The sample size was calculated separately for the two primary components of the study: (1) the before-and-after evaluation of GPs’ knowledge and (2) the comparison of patient biomarker outcomes between the intervention and control groups. For the knowledge assessment, the sample size was calculated using the G*Power (version 3.1.9.7; Heinrich-Heine-Universität Düsseldorf) based on the proportion of GP trainees enrolled in diabetes CME [44]. Assuming a power of 80%, a significance level of 0.05, and an effect size of 0.6, a total of 24 participants was sufficient to detect a significant difference in knowledge scores before and after completing the CME courses. For the patient outcome component, the sample size estimation was based on previously published research in which patients with diabetes received MI-guided behavioral change counseling [26]. To detect a 10% effect size with an alpha of .05 and a power of 80%, a total of 241 participants was required. This meant that 121 patients were needed for each of the study and control groups.

### Participants and Recruitment

**Figure 1.**
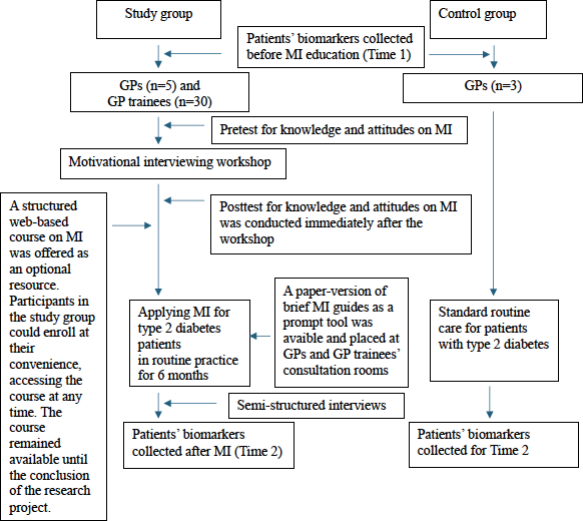
Flow of recruitment. GP: general practitioner; MI: motivational interviewing.

Figure 1 shows the flow of participant recruitment for the study. The study was conducted from January 2024 to January 2025.

#### GPs and GP trainees’ recruitment

Study and control groups were recruited by convenience from primary care units, which had family doctors working full-time. Notably, in Thailand, the majority of family or primary care doctors work in the hospitals, some work part-time at primary care units, and the majority of primary care units do not have doctors but are operated by nurse practitioners.

For the study group, a primary care unit affiliated with a medical school was selected. Doctors working at this unit included 5 full-time GPs and 2 to 3 GP trainees rotating in each month (the total number of GPs was 5, and the number of GP trainees was 30). For GP trainees, each would rotate at this primary care unit for at least 3 months per year. This primary care unit covered the care of 10 communities with a total population of approximately 13,000. For the control group, 3 primary care units were selected. These units served 4 communities with a combined population of approximately 10,897 and were staffed by 3 family doctors. Notably, the study and control areas are located approximately 300 km apart.

#### Patients’ Recruitment

The study used primary care catchment areas as the unit of allocation and analysis. This enabled the impact of the interventions to be examined at the population level and allowed the outcomes related to diabetes care for individuals to be examined, regardless of whether care was sought from a single or multiple GPs within their community. All patients with diabetes under care of the selected primary care units were included in the analysis as a unit of analysis. Therefore, 149 patients with diabetes in the study areas and 167 patients with diabetes in the control areas were included.

### Educational Interventions

An interactive 4-hour workshop on MI was offered to GPs and GP trainees who worked at the primary care unit of the study group. The instructors (IT and PN) attended several MI web-based courses provided by William Miller, Stephen Rollnick, and Theresa Moyers. The GPs and GP trainees voluntarily attended the workshop. The content of the workshop included human behavior, introduction to MI, roles of GPs in MI technique, MI spirit, 4 processes of MI, brief MI for busy GPs caring for people with diabetes, MI vs stage of change, and case studies using MI in each stage of change.

An additional educational course on MI was offered to GPs and GP trainees to self-study at their convenience. A structured web-based course on MI and stage of change was designed and developed to provide the foundation for GPs using MI in their routine consultations. The web-based course was developed and designed by incorporating several steps to create effective web-based learning activities, as recommended in a previous study [45]. The intervention, designed to provide GPs with knowledge of MI, was offered for a period of study. The content was designed as text-based plus interaction with text, that is, knowledge-based quizzes, self-practice, and self-reflection activities. The course comprised approximately 4 hours of learning activities for individuals to complete. The course content was developed in a manner similar to the workshop. Notably, participants could access other forms of MI education during the study period. The content of this course could be accessed through our medical school web-based platform free of charge.

A paper version of brief MI guides as a prompt tool for GPs to help people with diabetes change their behavior was available and placed in the GP consultation room at the primary care unit. These guides were developed based on “A Taste of MI” Exercise from the Motivational Interviewing–Foundational web-based course by William Miller, Stephen Rollnick, and Theresa Moyers (see the English translated version of brief MI guides in Multimedia Appendix 1).

### Quantitative Component

#### Data Collection: GPs and GP Trainees

A before-and-after design was used to compare the knowledge and attitudes of the GPs and GP trainees who completed the MI workshop. The GPs and GP trainees self-selected by voluntarily choosing to enroll in the workshop and the web-based course and participating in the before-and-after study. The questionnaires were developed by the authors (IT and RM) based on the literature [17] and inputs from academic GPs and experts in the field. Each item was assessed thoroughly regarding the intention of the measurement, relevance, ambiguity, understandability, and necessity by 3 independent experts in medical education and academic GPs at our medical school to ensure the face and content validity of the questionnaires. The tests were conducted on the web and divided into 2 phases: pre- and postsurveys. These tests included questions in the form of multiple-choice, yes-no, open-ended, attitudinal, and knowledge questions. The questionnaires were piloted with 30 GPs and were revised accordingly. Cronbach α coefficient of the questionnaire was 0.95.

#### Data Collection: Patient Outcomes

Data on patients with diabetes were collected twice from the patient data records. First, before the GPs and GP trainees attended the MI workshop. Second, 6 months after the workshop completion. This means that GPs and GP trainees had time to apply MI for their patients for at least 6 months. Patients’ biomarker data were obtained from the patients’ medical records for the latest values collected for each patient, according to the routine collection of the primary care units. Patient data included blood pressure, body weight, and HbA_1c_ and lipid levels.

### Qualitative Component—Data Collection: Interviews With GPs and GP Trainees

Semistructured interviews were also conducted. The GPs and GP trainees who completed the pretest were invited to participate in semistructured interviews. A small sample of 10 GPs and GP trainees was sought for convenience. A total of 5 GPs or GP trainees from each of the following groups were sought: GPs and GP trainees who reported having less confidence in applying MI in their routine consultation and those who reported having confidence in applying MI in their routine consultation. The interviews were conducted in September 2024 via Zoom (Zoom Communications) and were recorded for documentation. Each session lasted approximately 15 minutes. The audio recordings were transferred to a computer for repeated listening and detailed content review. The recordings were transcribed into Microsoft Word documents, and they were confirmed with participants to ensure accuracy and for further analysis.

### Quantitative Analysis

IBM SPSS 19 for Windows (version 20.0) was used for statistical analysis, and a pairwise deletion strategy was applied to handle missing data. Paired-sample *t* tests were applied to assess within-group differences between pre- and postintervention measurements, and independent-sample *t* tests were used to compare outcomes between the study and control groups. Baseline characteristics were descriptively compared across study and control groups; however, formal statistical adjustments for potential confounders (eg, baseline differences in patient demographics or clinical characteristics) were not performed. This decision was based on the study’s design focus on population-level comparisons and the available sample size, which limited the feasibility of multivariable analyses. Descriptive statistics were used to describe demographic data. The participants’ responses on Likert scales to a 10-item questionnaire on their confidence in applying MI for diabetes management were tallied, creating a confidence score range of 10 to 50. A paired-samples *t* test was used to compare the mean differences in the knowledge scores between the two tests.

### Qualitative Analysis

Interview transcripts were analyzed using conventional content analysis. An inductive coding approach was applied, whereby initial codes were generated from repeated reading of transcripts. These codes were then grouped into categories, and overarching themes were derived by identifying recurring patterns and relationships among the data. Coding and analysis were conducted manually by a single trained coder (IT). Although intercoder reliability was not assessed, the coding process was reviewed by members of the research team to enhance consistency and credibility. The codes were then organized into themes, identifying the relationships and connections between them.

### Integration of Quantitative and Qualitative Data

The quantitative and qualitative findings were synthesized during the interpretation phase to provide a comprehensive understanding of the intervention’s impact. Quantitative data provided objective evidence of improvements in knowledge and patient outcomes, while qualitative insights offered contextual understanding of how GPs applied MI, the challenges encountered, and their suggestions for improvement. This integration supported triangulation of results and enhanced the depth and relevance of the study’s conclusions.

### Ethical Considerations

This study was approved by the Human Research Ethics Committee of Khon Kaen University (project HE631031). GPs and GP trainees were recruited through the researcher’s assistant, who invited volunteers to participate. Before completing the questionnaires, participants were informed that participation was voluntary and that they could drop out of the study at any time. They were informed that their opinions were important for enhancing diabetes practice in primary care and were therefore encouraged to express them. All GPs and GP trainees voluntarily agreed to participate without receiving compensation. The participants’ privacy and identity were protected, and confidentiality was assured. Informed consent was obtained from each participant who participated in the semi-structured interviews. The study objectives were explained to the participants and the study was conducted according to the academic ethical code.

For patients, individual consent was not required because all clinical outcome data were extracted from anonymized, routinely collected health records, in accordance with institutional ethical guidelines. No experimental procedures or additional data collection were imposed on patients. After the study concluded, the brief MI materials and web-based MI course were shared and offered to clinicians in the control group as part of standard CPD dissemination. This approach ensured that no participants were denied care and aligns with accepted ethical principles for real-world implementation research.

## Results

### Participants’ Characteristics

The MI workshop was attended by 32 GPs and GP trainees out of a total 35 participants (91%). A total of 2 participants out of 38 had previous training in MI (n=2, 5%). While both GPs and GP trainees participated in the educational interventions (n=32), only 28 GP trainees (87%) completed the pre- and posttests to assess their knowledge, and later 10 GP trainees provided feedback through semistructured interviews, whereas no GPs participated in these assessments (see Table 1).

**Table 1.** Participating general practitioners (GPs) and GP trainees’ demographic data.

Demographic data (N=38)^a^	Results
Sex, n (%)	
Males	18 (47)
Females	20 (53)
Age (years)	
Mean (SD)	28.5 (7.85)
Median (range)	28 (25-62)
Previous web-based learning, n (%)	
Yes	25 (66)
No	13 (34)
Previous web-based course completion	
Mean (SD)	0.86 (1.15)
Median (range)	1 (0-4)
Previous motivational interviewing course completion, n (%)	
Yes	2 (5)^b^
No	36 (95)
Diabetes patients in the catchment areas, n (%)	
Study areas (N=13,000)	149 (1.14)
Control areas (N=10,897)	167 (1.53)

a A total of 35 GPs and GP trainees participated in the study group, while 3 were from the control group.

b The general practitioners in the study group.

Data from the web-based learning management system (LMS; (updated on January 21, 2025) showed that there were 35 GPs and GP trainees (100%) enrolled in the structured web-based MI courses, 14/35 (40%) completed the courses, 5/35 (14%) had not yet started the lesson, and 16/35 (46%) had started but had not yet completed. The course had 21 lessons. Participants’ enrollment time in the course was a median of 3 minutes (range 0-416 min), with a mean of 68.54 (SD 108.82). The number of completed lessons by participants was a median of 2 lessons (range 0-21), with a mean of 6.46 (SD 8.79).

### Changes in MI Knowledge and Confidence

A paired-sample *t* test was conducted to evaluate the impact of the MI course on learners’ knowledge. There was a statistically significant improvement in knowledge test scores from Time 1 (mean 11.46, SD 3.48) to Time 2 (mean 15.04, SD 2.35), *t*_28_=7.74; *P*<.001 (2-tailed). The mean increase in knowledge test score was 3.57 (SD 2.44), 95% CI 2.62-4.52. Notably, overlapping pre- and postintervention CIs do not invalidate the finding, as this was a within-subjects comparison. The eta-squared statistic indicated a large effect size (eta-squared=0.85). Table 2 shows participants’ confidence levels in applying MI techniques after completing the course. The highest confidence was reported for the focusing process (mean 3.86, SD 0.65) and using affirmations (mean 3.79, SD 0.63). Other skills, such as using open-ended questions, planning, and inviting change talks, scored slightly lower but consistently reflected moderate to high confidence levels, indicating the course’s effectiveness in enhancing practical MI skills.

**Table 2.** After the course completion, participants’ level of confidence in applying motivational interviewing (MI) with patients (n=28).

Participants’ level of confidence in applying MI with patients	Mean^a^ (SD)
Focusing process	3.86 (0.65)
Using affirmation	3.79 (0.63)
Using open-ended questions	3.71 (0.76)
Planning process	3.71 (0.66)
Inviting change talks	3.68 (0.61)
Using summary	3.68 (0.67)
Using MI with stage of change	3.68 (0.61)
Using reflection	3.61 (0.63)
Responding to change talks	3.61 (0.74)
Recognizing change talks and sustain talks	3.57 (0.63)

a Mean was calculated using a 5-point Likert scale ranging from 1 (not at all confident), 2 (somewhat not confident), 3 (neutral), 4 (confident), and 5 (very confident).

### Changes in Patient’s Outcomes

Table 3 provides biomarker outcomes for patients with diabetes over 6 months after GP trainees completed the MI workshop. In the study group, there were significant improvements, including reductions in HbA_1c_ (mean difference 0.35, 95% CI 0.03-0.66; *P*=.03), body weight (mean difference 1.11, 95% CI 0.27-1.95; *P*=.01), triglyceride (mean difference 14.18, 95% CI 1.69-26.67; *P*=.02), systolic blood pressure (mean difference 5.14, 95% CI 1.06-9.03; *P*=.01) and diastolic blood pressure (mean difference 3.82, 95% CI 1.66-5.99; *P*=.001). Between-group analysis showed that the study group had significantly better outcomes for HbA_1c_ (mean difference −0.50, 95% CI −0.91 to −0.09; *P*=.02) and diastolic blood pressure levels (mean difference −5.96, 95% CI −8.66 to −3.25; *P*<.001) compared to the control group.

**Table 3.** The patients’ biomarkers before and after 6 months of GPs and GP trainees’ enrollment in the motivational interviewing (MI) workshop. The number of cases used in each calculation varies slightly across variables.

Patient outcomes	Study group (n=149)					Control group (n=167)					Between group			
	Pre- MI mean (SD)	Post-MI mean (SD)	Mean difference (SD)	95% CI	*P* value (within group)^a^	Time 1 mean (SD)	Time 2 mean (SD)	Mean difference (SD)	95% CI	*P* value (within group)^a^	Pre-MI		Post-MI	
											Mean difference (95% CI)	*P* value^b^	Mean difference (95% CI)	*P* value^b^
HbA_1c_	8.07 (1.59)	7.71 (1.53)	0.35 (1.88)	0.03- 0.66	.03	8.38 (1.97)	8.23 (2.10)	0.16 (1.74)	−.11 to .42	.25	−0.30 (−0.70 to 0.11)	.11	−0.50 (−0.91 to −0.09)	.02
Body weight	63.55 (12.33)	62.44 (12.89)	1.11 (4.70)	0.27- 1.95	.01	59.83 (11.51)	58.41 (11.71)	1.42 (5.08)	0.64-2.20	<.001	3.08 (0.49-5.68)	.02	4.03 (1.16-6.89)	.006
Systolic blood pressure	138.94 (18.47)	133.79 (18.55)	5.14 (23.40)	1.06- 9.03	.01	125.69 (12.59)	131.32 (14.33)	−5.62 (16.05)	−8.09 to −3.15	<.001	13.16 (9.60-16.73)	<.001	2.47 (−1.22 to 6.17)	.18
Diastolic blood pressure	74.32 (11.61)	70.50 (11.06)	3.82 (13.04)	1.66- 5.99	.001	73.02 (8.88)	76.46 (12.74)	−3.43 (14.54)	−5.67 to −1.20	.003	1.13 (−1.18 to 3.45)	.33	−5.96 (−8.66 to−3.25)	<.001
Cholesterol	181.21 (37.66)	179.33 (43.69)	1.89 (40.66)	−5.11 to 8.88	.59	177.26 (41.60)	155.38 (37.49)	21.87 (35.19)	16.46-27.28	<.001	4.81 (−4.08 to 13.71)	.28	23.62 (14.47-32.77)	<.001
Triglyceride	154.98 (81.19)	140.79 (69.61)	14.18 (72.26)	1.69- 26.67	.02	181.12 (124.59)	151.38 (72.65)	29.73 (119.01)	11.44-48.03	.002	−27.27 (−50.13 to −4.42)	.02	−10.35 (−26.62 to 5.90)	.21
LDL^c^	107.58 (33.43)	107.04 (37.37)	0.54 (38.61)	−6.03 to 7.11	.87	105.25 (31.97)	93.26 (31.26)	11.99 (30.27)	7.33-16.64	<.001	4.00 (−3.46 to 11.47)	.29	13.43 (5.49-21.36)	<.001
HDL^d^	51.40 (13.60)	51.92 (13.03)	−0.51 (9.02)	−2.07 to 1.04	.52	51.21 (13.22)	50.04 (13.12)	1.17 (11.56)	−0.60 to 2.94	.19	0.17 (−2.80 to 3.15)	.90	1.82 (−1.15 to 4.81)	.22

a Data were analyzed using paired-samples *t* test.

b Data were analyzed using independent-samples *t* test.

c LDL: low-density lipoprotein.

d HDL: high-density lipoprotein.

### Learners’ Experiences and Perceived Impact of the Course

Table 4 summarizes the participants’ expectations before enrolling in the MI workshop and how these expectations were met after the workshop. Participants reported improvement in skills and knowledge about MI, with mean scores increasing from 3.96 to 4.07 and from 3.93 to 4.14, respectively. In addition, the learning methods, venue, and learning period received positive ratings postcourse, highlighting the course’s effectiveness in meeting expectations.

**Table 4.** Expectations for learning in this motivational interviewing (MI) course before and how they were met after the participants’ enrollment in the MI workshop (n=28).

Participants’ expectations before enrolling in MI courses and reported their expectations were met after the course completion	Mean^a^ (SD)	
	Before enrollment in the course	After the course completion
Having skills in MI	3.96 (0.69)	4.07 (0.86)
Having knowledge about MI	3.93 (0.60)	4.14 (0.80)
Promptly applying MI techniques with patients	3.64 (0.83)	3.79 (0.79)
After applying MI with patients, the patients can control their diseases	3.93 (0.77)	—^b^
After applying MI with patients, the patients can change their behaviors	3.96 (0.79)	—
Teaching and learning methods for MI	—	3.93 (0.77)
Place or venue for learning	—	3.96 (0.88)
Period of learning	—	3.86 (0.89)

a Mean was calculated using a 5-point Likert scale ranging from 1 (strongly disagree), 2 (disagree), 3 (neutral), 4 (agree), and 5 (strongly agree).

b Not available.

### Insights From Participant Interviews

Table 5 summarizes the interviews with participating GP trainees regarding their experience in implementing MI during their routine consultation. The GP trainees found MI useful for engaging patients with diabetes, especially in brief MI formats.

All participants reported that MI techniques were used in patient interactions, particularly in cases of diabetes, hypertension, and dyslipidemia. The engaging and evoking processes were most frequently used, while planning and responding to sustain talk were less commonly applied.

**Table 5.** General practitioner (GP) trainees’ experiences and challenges in implementing motivational interviewing (MI) in their routine consultation.

General practice trainees’ experiences in and perceptions toward the use of MI with patients	Comments
Experience with applying MI in practice. Contexts and types of cases where MI is used.	
All participants reported using MI techniques in their patient interactions. They primarily applied MI in cases involving noncommunicable diseases, such as diabetes, hypertension, and dyslipidemia. In addition, all participants attempted to use brief MI, particularly in diabetes cases (T1-T5^a^ and P1-P5^b^).	“Most of the cases I applied MI are NCDs” (T3). “I tried to apply MI with my patients to help them with lifestyle modification, including for NCDs, diabetes, hypertension, dyslipidemia, but just some processes of MI such as empathic listening” (P5). “I have only experienced using brief MI as a guide for approaching patients, and all the cases were diabetes, so I have not applied the full MI process with my patients” (T5). “While I was rotating in Internal Medicine, I applied MI with patients to help them lose weight and with the issues of nutrition management. During my rotation at primary care practice, I used brief MI for only diabetes patients” (T1).
Most frequently used MI processes (eg, engaging, focusing, evoking, and planning)	
For full MI, the majority of participants most frequently used the engaging process, as they were familiar with its fundamental skills, such as open-ended questions, affirmations, reflections, and summaries (T2-T3 and P2-P5). A participant reported primarily using the focusing process (T5), while others frequently applied the evoking process (T1, T4, and P1). For those using brief MI, all MI processes were experienced; however, they might not have explicitly recognized the distinct processes involved (T1-T5 and P1-P5).	“Engaging is easy to use. It is like a small talk before starting the consultation. I used open-ended questions quite often as well as reflection” (P3). “I used only brief MI with all diabetes cases,… engaging with fundamental skills and evoking with important ruler” (P2). “Evoking with important ruler is easy to use” (T1). “Engaging since it is like we are getting to know the patient like a small talk before starting consultation, so I’m familiar with OARS and open-ended questions were used the most” (P4).
Least used MI processes and reasons behind it.	
The evoking process (T3, T5, and P2-P5) and the planning process (T1, T2, and T4) were reported as the least frequently used by participants. The primary reasons included not yet reaching these stages in their patient interactions, uncertainty about how to transition to these processes, or a lack of opportunity to follow-up with patients until the final stage of MI due to rotations in their training programs.	“I am not yet good at using evoking and also planning since I have not yet took the patient to this process yet and I did not have a chance to follow-up the same patient because of changing rotation” (T3). “Planning process was used the least since I have not reached this process yet and sometimes was not sure how to go next” (T1). “I have not reached this process yet and I am not familiar of how to use it” (T2). “Evoking is the least. I tried to use important ruler to invite change talks however, it seemed like patient did not understand particularly those aged above 60.” (T5). “I tried to all the processes, but each was like little here and there” (P1) “I’m not yet familiar with evoking. To start evoking, you may need to have good relationship with patients and important ruler is hard to understand” (P4).
Challenges faced when applying specific MI processes	
All the participants reported that evoking process is the most difficult to use in all aspects of evoking including recognizing, inviting, and responding to change talks (T1-T5 and P1-P5).	“To deal with sustain talk, how to respond to sustain talk is the most difficult for me” (T1). “Evoking, particularly inviting patient for change talk, it is difficult to do” (P1). “In inviting change talks, patient did not understand the question, particularly using important rulers and it felt like it was not flow naturally” (T3). “Evoking is difficult particularly, recognizing and inviting change talks since I do not have much experience in applying full MI sessions but for brief MI, there is check lists to help how to proceed” (P2). “Recognizing change talk is the hardest part because it’s difficult to identify which statements are actually change talk. In some cases, patients didn’t say much, and when they did, their speech was not sequential, making it hard to distinguish between change talk and sustain talk” (P4).
Obstacles encountered during the use of MI in routine consultations	
All participants (T1-T5 and P1-P5) agreed that incorporating MI into routine consultations adds extra time to each patient encounter. They noted that if the daily patient load were lower, MI could be applied more comfortably. While brief MI may offer a practical solution, participants acknowledged the need to first master the technique to use it effectively without extending consultation time. Another challenge is limited experience, which hinders skill development and prevents MI from becoming a natural part of routine practice. In addition, for GP trainees, structured training rotations often limit their ability to follow-up with patients through the full MI process.	“Time and number of patients seeing in routing consultation per day. If small number of patients like if less than 60 a day, MI can be used but if not, it would increase time for each case and ended up with delay waiting time for the next patient” (P2). “Elderly patients do not understand the questions may be because doctor could not make the question flow naturally or even do not know how to make it easy to answer. I, myself, not yet good at execute MI” (P2). “MI takes longer consultation time. It matches with the setting that have a smaller number of patients or have many doctors in the same practice or it needs to separate MI session out of routine consultation, but this would be solved with brief MI” (T4). “I used brief MI, it helps a lot but since I’ m not yet mastered the whole processes of MI, so it is not yet felt naturally in applying it routinely” (P5). “Because of trainee rotation, I cannot follow the same patient till the final process of MI” (T1). “I have less experience in using MI but when start using it I feel good and better when dealing with patient’s lifestyle modification, so I need to practice more” (T1). “In the form of brief MI, it helps shorten the time but overall, still adds time to the consultation and my MI skills are not yet ready” (T3). “MI needs time to explore patients to get to know them and sometimes they did not know how to respond or say, so it takes time” (P3). “Spending longer time and together with “I’m not yet good at using MI,” sometimes I need to stop thinking what is next because this also adds up time” (P4).
Perceived benefits of using MI in routine consultations	
All participants (T1-T5 and P1-P5) highlighted the advantages of MI, emphasizing its foundation in the patient-centered approach. They noted that MI empowers patients to decide what and how to change, while the doctor provides guidance and support to help them achieve their goals.	“Patient selects by themselves what they want to change so this will eventually lead to actual change” (T2). “Concepts and principles of MI are great, unlike the traditional approach of ordering or telling patients to change but MI is to guide and help them change with the direction” (T3). “We as a doctor understand patient more and respect them for whom they are” (T4). “There is a direction in MI, making it like a framework that guides us toward the goal. The process consists of steps or pathways that help us navigate patient interactions and continue follow-ups from where we left off in the last visit. Otherwise, we would end up repeating the same advice at each visit, providing a generic package of behavior change recommendations, which would ultimately lead to unsuccessful outcomes.” (P2).
Potential drawbacks or limitations of using MI in consultations	
Participants identified several limitations in implementing MI in routine consultations. The primary challenge was a lack of skill, largely due to limited experience in applying MI in daily practice (P1-P3). While brief MI offers a promising approach, it still requires practice to master and integrate naturally into consultations without extending their duration (T1, T4, and T5). Since participants had not yet fully mastered MI, their consultations often took longer than usual (P1 and P2).	“Full MI taking long time and may not suit particularly in the setting of outpatients where each doctor needs to see 100 cases a day” (T5) “If you are not familiar or not good at it, it will take more time in consultation. In some cases, we cannot use this method such as dementia” (P2). “I’m not yet confident in using the MI process. Those who want to apply MI should have adequate knowledge and skills, become familiar with the process, and integrate it into their routine practice” (P3). “I could not yet find any limitation or disadvantage” (T3) “Take longer time in consultation and need to be cautious using some questions which may affect the doctor-patient relationship” (P4).
Areas for further development in MI	
Some participants need to improve all knowledge and skills to gain their confidence in using MI (T2-T5, P4, and P5). Some mentioned have sufficient knowledge but need more practice to improve skills and particularly for evoking process (P1-P3).	“I feel a lack of knowledge and skills in MI. I think I know and can do only 50%. Brief MI works for me — no need for more time in consultation. It is practical, easy to use, and can be applied in real practice” (Participant 5). “Knowledge and skills in evoking are still limited, and I also have little experience using MI. Sometimes I have to focus too much when using MI, as I need to practice empathic listening, be able to reflect back what the patient is saying, and also summarize parts of the conversation” (P4). “Since I have not yet become good at using MI and do not use it regularly, only with some diabetes patients using brief MI, I need more practice to build my confidence” (P3). “My knowledge is ok but I need to improve my skills, particularly for evoking process” (P2). “I am not yet confident in my overall MI knowledge and skills, so I can not apply them naturally in practice. In the evoking process, for inviting change talk, there are plenty of ways to do it, so I need to go back and practice more of those options” (T5). “I need to update all MI knowledge and skills” (T4).
Need for additional training or support in MI techniques	
Majority of the participants need more observations to become experts in using MI in routine practice and, if possible, need coaching (T1, T3-T5, and P2-P5). One mentioned role-play sessions with colleagues will also help to gain more confidence in applying MI in their routine consultation (P1). Additional video clips would also help in self-learning (P1). Others needed to practice brief MI more (T2 and P2).	“I need to keep practicing using MI in my routine consultations, and share cases that were successful after using MI. I also need observation sessions with experts or supervisors, as well as coaching” (P4). “I need more observation sessions to see experts using this technique in real practice, followed by coaching to get feedback on whether I’m doing it right” (P3). “I need more role plays in class so all my colleagues can practice together and learn which parts we do right or wrong, and practice the whole process step by step. Other helpful methods would be coaching or observation in real practice settings. Video clips of different situations applying MI in routine consultations would also be great” (P1). “I want more observation from expert or coaching” (T3) “I need to practice brief MI more and use more” (T2) “I need more examples of how to use MI in a variety of situations—what to say and how to say it, and how to apply MI in each situation. These would help me get a clearer picture of applying MI in real consultations. Brief MI is great and I can use it in my routine, but I need more observation classes to use it naturally rather than relying on checklists or guidelines all the time” (P2).
Impact of MI on practice and likelihood of continued use	
Initially, MI has changed their practice, getting to know more for their own patients. All participants mentioned further practice in MI and will continue using it in their routine consultation (T1-T5, P1-P5).	“I will definitely continue to use MI in my routine practice, particularly brief MI, as it does not take more time than a regular consultation. I will use it as part of my routine work because even when there are many patients, brief MI can still be applied within 5‐6 minutes as part of usual care” (P2). “There are many advantages to applying MI in routine consultations, particularly for DM and CKD patients, since these techniques can be used before they become sick, while they are sick, and also in the process of tertiary prevention for disease complications” (T5). “I will definitely continue practicing until I master MI and will integrate it into my routine practice” (P5). “The process of MI is very interesting; it helps you have something to talk about with patients and understand them more. I need more practice to become good at it and use it in my routine practice” (T4). “Empathic listening and important rulers help you get clear answers and lead to change” (T2). “I will use it more, since if the patient selects the option themselves—what and how to change—it is much better than having someone else tell them what to change” (T1). “I will definitely continue using MI so the way I help my patient with their lifestyle change is now not the same” (T3).

a T: Participants who reported having confidence in applying MI in routine consultations, based on pretest survey data.

b P: Participants who reported having less confidence in applying MI in routine consultations.

*I feel confident using open-ended questions and affirmations because they make patients open up more, and I can understand their situation better.*

[P3]

*Even with brief MI, I tried to engage and ask about their goals… patients seem more open, and it helps me decide what to suggest next.*

[T2]

However, many participants noted difficulty in applying the evoking and planning processes.

*I know the steps of MI, but the evoking part—like using importance rulers or asking about change—is really hard to apply when the patient doesn’t understand the question.*

[T4]

*Planning is hard when we don’t see the patient again. During rotations, we only see them once, so it’s difficult to finish the full MI cycle.*

[P5]

Challenges also included time constraints and unfamiliarity with the MI approach.

*MI takes longer time in consultation and my skills are not yet good enough, so I need to stop and think before responding.*

[P1]

Despite these obstacles, participants valued the patient-centered nature of MI.

*Using MI makes me feel like I really listen to my patients. They appreciate it more, and some of them even said they feel more motivated to change.*

[T1].

*Brief MI is a practical tool I can use in real life—it gives structure to the conversation without taking too much time.*

[P2].

## Discussion

### Principal Findings

This study evaluated the implementation of a combined educational intervention on MI for GPs and GP trainees involved in type 2 diabetes care. The findings revealed significant improvements in participants’ MI knowledge, confidence, and related patient outcomes. Furthermore, participants reported positive experiences with the training and perceived enhancements in their communication skills and patient care practices.

All GPs and GP trainees in the study group completed the interactive MI workshop, but only GP trainees participated in knowledge pre- and posttests, and the latter half of GP trainees completed the structured web-based courses. The study demonstrated that MI education provided for GPs and GP trainees in interactive workshop formats significantly improved their knowledge, and together with additional self-learning through structured web-based courses and brief MI guides, had a measurable impact on the patient outcomes. In terms of patient outcomes, the MI-trained group showed improvements in key health metrics, such as HbA_1c_ and diastolic blood pressure, compared to the control group, emphasizing the practical benefits of MI training in clinical settings. Semistructured interviews revealed that GP trainees valued MI for its patient-centered approach but faced challenges related to its time demands and their own proficiency in its processes. However, this study was conducted in a single study area; therefore, the results should be interpreted with caution.

This study contributes to the literature on CME and the application of behavioral science in clinical practice. It addresses the gap between theoretical knowledge of MI and its practical application by GPs and trainees in routine care. Through the design of a structured, multifaceted CME intervention—comprising an interactive workshop, web-based learning, and practical tools—this study illustrates how behavioral science principles can be effectively translated into real-world practice. The findings may also guide the development of future training programs to enhance patient-centered communication among healthcare providers.

### Effects of MI Training on GP Trainees’ Knowledge and Confidence

GP trainees who underwent MI training demonstrated a significant improvement in their knowledge scores. The educational program successfully met its objective of enhancing knowledge among GP participants, aligning with previous studies that highlight the potential of MI education to improve both knowledge and confidence in delivering behavior-change interventions [17,31].

Knowledge assessments were conducted before and immediately after the workshop, indicating an immediate knowledge gain. This finding is consistent with previous studies on the effectiveness of MI training in primary care, which have demonstrated improvements in both knowledge acquisition and skill application [17,46,47]. Given that knowledge was assessed immediately after the workshop, the observed improvement could be attributed primarily to the interactive workshop component. However, following the workshop, participants also had access to web-based resources and MI guides. The combination of these educational methods aligns with existing evidence demonstrating that multifaceted educational interventions effectively enhance not only knowledge but also clinical practices and patient outcomes [34,35].

The educational content covered MI processes and their practical application through case studies in accordance with the frameworks established by Miller and Rollnick [11]. Notably, all participants who took the knowledge pre- and posttests had no previous MI training, which aligned with their self-reported learning expectations from the pretraining questionnaire. This targeted approach contributed to the successful development and implementation of an effective educational program as it addressed learners’ specific needs [44].

Participants’ feedback indicated that fundamental MI processes such as engaging, evoking, and planning were effectively taught. However, more advanced skills, particularly responding to change talk, remain challenging, an issue frequently noted in MI training [30,48]. Participants also reported that this was their first exposure to MI, and that the combination of workshop-based learning, structured web-based modules, and brief MI guides was sufficient for acquiring foundational MI knowledge.

In addition to knowledge acquisition, the training significantly enhanced participants’ confidence in applying MI techniques, particularly in the practical aspects of brief MI. As shown in Table 3, participants reported the highest confidence in performing the focusing process and using affirmations, both essential elements of patient-centered communication. Other MI skills—including open-ended questioning, planning, and inviting change talks—also received moderate to high confidence ratings. These findings support the effectiveness of the combined educational interventions in building participants’ self-efficacy for using MI in clinical practice. The pattern of reported confidence aligns with the stages of skill development, where foundational processes such as engaging and focusing are typically mastered earlier, while more advanced tasks like evoking and planning require further practice and reinforcement. This confidence boost is crucial, as self-efficacy is strongly linked to successful behavioral change counseling and continued use of MI techniques in routine care.

### Impact of MI Training on the Patient Outcomes

Patient outcomes in this study were analyzed at the unit level. Patients with diabetes treated by MI-trained GPs and GP trainees demonstrated significant improvements in HbA_1c_ levels, body weight, triglyceride levels, systolic blood pressure, and diastolic blood pressure when comparing pre- and posteducational interventions and MI implementation. However, only HbA_1c_ levels and diastolic blood pressure showed statistically significant differences between the study and control groups after the educational interventions and MI implementation. Notably, although the study was powered to detect a 10% change in key clinical outcomes, the observed changes in most patient biomarkers—including HbA_1c_, blood pressure, and lipid levels—were smaller than this threshold. While some changes reached statistical significance, the study may have been underpowered to detect modest effects, and tests of significance should be interpreted with caution. These findings suggest that knowledge gained from a single training session, supplemented by self-learning through the provided resources, can be effectively applied in clinical practice, leading to measurable improvements in diabetes patient outcomes.

Although few studies have examined MI delivered by GPs for diabetes patient outcomes, this study aligns with previous research demonstrating MI’s mixed but promising efficacy in GP-led diabetes management [17]. Similar to the current findings, previous studies have reported improvements in HbA_1c_ levels between the study and control groups [49,50], and reductions in diastolic blood pressure [49].

This study found improvements in systolic blood pressure within both groups, but no statistically significant difference between them, which is consistent with previous findings [51]. A similar pattern was observed for triglyceride levels [51]. Notably, the study also found a positive effect on body weight in both groups; however, the control group showed a statistically greater reduction than the study group. In contrast, a previous study reported no significant impact on body weight [52]. Despite the variability in effects of MI on patient outcomes, these findings contribute to a growing body of evidence supporting the potential of MI when implemented by GPs in diabetes care.

Semistructured interviews with GP trainees in this study reinforced the quantitative findings, as participants reported that MI facilitated better patient engagement and empowered individuals to make sustainable behavioral changes. Although many GP trainees did not complete all 4 MI processes with patients, the majority implemented brief MI guides during diabetes consultations. Participants most frequently applied the engaging and evoking processes, which may help explain the observed improvements in HbA_1c_ and diastolic blood pressure—both outcomes known to be influenced by enhanced patient motivation and communication. Even limited MI skills, such as empathic listening and goal-focused dialog, have been perceived as effective in fostering patient engagement, supporting the feasibility of brief MI formats in high-volume primary care settings. However, some clinical metrics, such as triglyceride levels and systolic blood pressure, have shown mixed results, highlighting the need for further research on MI uptake and its long-term impact, particularly regarding the sustained effects of brief interventions. This variability aligns with a systematic review that identified mixed yet promising results in MI interventions, underscoring the importance of more consistent training and implementation to improve outcomes [17].

While the observed improvements in HbA_1c_ and diastolic blood pressure among patients in the MI-trained group are promising, it is important to interpret these findings with caution. HbA_1c_ is a valid and widely used clinical outcome; however, it can be influenced by a variety of factors beyond the educational intervention, such as medication adherence, lifestyle changes, appointment attendance, or changes in health service delivery. Since we did not measure intermediate outcomes such as patient adherence, satisfaction, or visit frequency, the attribution of clinical improvements solely to MI training may be limited. Future studies should incorporate additional process measures to more fully explore the mechanisms by which MI training may impact patient outcomes. We also acknowledge limitations related to the interpretation of lipid profile outcomes. Variability in LDL and total cholesterol levels across groups may reflect unmeasured factors such as baseline statin use, medication adherence, and dietary or genetic differences. These variables were not adjusted for in this analysis. We recommend that future research include medication adherence monitoring and baseline lipid-lowering therapy documentation to better account for such confounders in lipid-related outcomes.

In addition to statistical significance, the improvements observed in certain outcomes appear to be clinically meaningful. Patients managed by MI-trained GPs and GP trainees demonstrated significant reductions in HbA_1c_ and diastolic blood pressure compared to the control group. These changes are important in diabetes management, as even modest reductions in HbA_1c_ and blood pressure have been linked to decreased risk of microvascular and cardiovascular complications. While improvements in other parameters, such as systolic blood pressure and triglycerides, were mixed or nonsignificant between groups, the observed HbA_1c_ and diastolic blood pressure reductions suggest that gains in MI-related knowledge and confidence may have translated into tangible patient benefits. This underscores the potential for brief MI training, when implemented in routine primary care, to yield clinically relevant outcomes.

### GP Trainees’ Perspectives and Experiencers Implementing MI

MI requires complex skills [53]. Effective MI implementation necessitates comprehensive training programs that integrate didactic presentations, experiential exercises, and role-playing to develop both basic and advanced MI skills. This study used these combined educational methods. In addition, these educational methods encourage practitioners to progress toward becoming trainers themselves [54]. Evidence suggests that practical applications and expert feedback are crucial for developing MI proficiency [46]. Participants in this study expressed a strong need for coaching or observational support from experts who could demonstrate MI in real consultations, aligning with research indicating that mentorship significantly enhances MI skills [55].

The GPs and GP trainees in this study were trained in the full MI process for comprehensive consultations. However, time constraints in Thai primary care settings make full-session MI difficult to implement. This issue has been noted in other health care settings, where time limitations hinder MI adoption [17]. To address this, brief MI guides were introduced, enabling GPs and GP trainees to apply MI techniques to patients with diabetes within typical consultation durations. Previous studies have suggested that brief MI can be an effective alternative in high-volume practices while maintaining its patient-centered benefits [30]. Participants from this study who had foundational MI knowledge from the workshop found brief MI guides particularly useful for integrating MI into clinical practice, aligning with research that highlights the importance of initial training before adopting streamlined approaches [26].

Qualitative data revealed mixed experiences with the implementation of MI. While GP trainees valued MI’s structured, patient-centered approach, they faced barriers such as time constraints, difficulty in mastering advanced techniques, and limited follow-up opportunities due to rotational training schedules. The engaging and evoking phases were widely used, but many GP trainees struggled with the planning phase, a challenge documented in previous MI research [17]. Participants also noted that MI’s emphasis on empathic listening and reflective questioning extended consultation times, making it difficult to sustain in high-volume clinical settings [30]. Although brief MI guides were designed to fit within standard consultation durations, some GP trainees reported that a lack of confidence in advanced MI techniques and incomplete mastery of MI skills could inadvertently prolong consultations. This aligns with findings from previous studies, which highlight that practitioners in the early MI training phases may take longer to implement MI effectively [47,56].

Despite these challenges, all participants acknowledged the benefits of MI, particularly brief MI, which they found more manageable within their workloads. Most reported using brief MI in nearly all encounters with patients with diabetes, while recognizing the need for continued practice to enhance their confidence and further refine their skills.

Our findings align with previous literature that supports the potential of MI to improve clinical outcomes in patients with type 2 diabetes when delivered by primary care providers [17,49,50]. Similar to previous studies, we observed a significant reduction in HbA_1c_ and diastolic blood pressure following MI training [17,49], reinforcing the value of even brief MI interventions when embedded in routine consultations. However, as reported in earlier work [17,30,48], our participants also faced challenges in mastering advanced MI processes such as evoking and planning. This underscores the need for structured, staged training that starts with foundational MI concepts and provides opportunities for progressive skill development.

In line with recommendations from systematic reviews on effective MI training [13,53,55], our study supports the implementation of multifaceted educational strategies. These include combining in-person workshops with asynchronous web-based learning, distributing practical tools (eg, brief MI guides), and offering ongoing peer coaching or expert feedback. Such approaches help accommodate varying learning styles and time constraints, particularly in high-volume clinical settings. Based on our findings, we recommend that MI training programs for GPs incorporate brief MI frameworks early in the training, followed by reinforcement through mentorship, peer observation, or reflective practice sessions to sustain skill application over time. Tailoring training to local practice constraints—such as patient volume, consultation duration, and provider rotation—can enhance feasibility and long-term adoption.

### Strengths and Weaknesses

This study has several strengths and limitations. The study design combining quantitative and qualitative approaches enriched the understanding of MI’s implementation in real-world practice and its impact on GPs and their patients, offering both measurable outcomes and contextual insights. The study’s focus on routine practice settings enhances the relevance and applicability of the findings to primary care environments. In addition, the pre- and posttraining assessments provided robust evidence of the effectiveness of training in improving knowledge of GPs.

While this study incorporated a control group to compare patient outcomes, we acknowledge that the nonrandomized design limits the ability to draw strong causal inferences regarding the effectiveness of the MI intervention. The control group consisted of primary care units in another province where GPs did not receive MI training and thus served as a comparator for usual care. Although this allowed for between-group comparisons, differences in context, provider characteristics, or patient demographics may have introduced selection bias or residual confounding. To minimize these biases, control sites were selected based on similarities in service structure, population size, and staffing (ie, family doctors in community-based settings). In addition, the use of population-level data from primary care catchment areas helped reduce provider-specific effects and reflect routine care delivery more accurately. However, unmeasured differences—such as provider motivation, local health system factors, or patient case mix—may have influenced the results. Therefore, findings should be interpreted with caution and viewed as indicative of association rather than definitive evidence of causality. Future studies employing randomized controlled designs or matched comparison groups with standardized training exposures and comparing the observed changes with broader regional trends in diabetes outcomes are recommended to strengthen causal conclusions and improve generalizability.

This study has many limitations. First, the study did not include a control group among GPs or GP trainees for the educational intervention, limiting our ability to attribute changes in learning outcomes solely to the MI training. Comparisons were made only within the intervention group before and after the training. Second, the sample size of GP trainees who completed both pre- and postassessments was modest, which may affect statistical power and limit the robustness of subgroup analyses. Third, the use of self-reported measures for MI confidence and qualitative interviews introduces potential bias, as participants may have overestimated their skills or provided socially desirable responses. Fourth, the relatively short follow-up period of 6 months for assessing clinical outcomes such as HbA_1c_ and blood pressure. While this timeframe allowed us to evaluate initial effects of MI training and implementation, it may be insufficient to capture longer-term behavioral change and sustained clinical benefits. In addition, while the study demonstrated statistically significant improvements in certain diabetes outcomes (eg, HbA_1c_ and diastolic blood pressure), the magnitude of these changes was modest. This may be due in part to the relatively short duration and intensity of the MI training provided. A single 4-hour workshop, even when supplemented by optional web-based modules, may not be sufficient to fully develop and sustain advanced MI competencies. Furthermore, qualitative findings indicated that GPs and trainees faced several implementation barriers, including time constraints, limited patient continuity, and lack of confidence in using more advanced MI processes such as evoking and planning. These challenges likely impacted the consistency and depth of MI delivery. Fifth, despite having a control group of patients with diabetes, the study sample consisted of a homogeneous group of GPs, GP trainees, and patients from a single area, potentially limiting the generalizability of the findings to broader populations. Another limitation is that no formal statistical adjustments were made for potential confounders, such as baseline differences in patient characteristics between the study and control groups. Although descriptive comparisons were conducted, the absence of adjusted analyses means residual confounding cannot be ruled out. Future studies using larger sample sizes and multivariable analytical approaches are warranted to strengthen the robustness of causal inferences. Another limitation is the absence of data on intermediate behavioral indicators—such as patient adherence to lifestyle recommendations, medication compliance, frequency of follow-up visits, or satisfaction with care—which may mediate or moderate the observed effects on HbA_1c_ and blood pressure. Including such measures in future studies would provide a more comprehensive understanding of the impact and mechanisms of MI in real-world primary care settings. Sixth, a further limitation relates to the qualitative analysis process. Thematic derivation was based on conventional content analysis using an inductive coding approach, whereby initial codes were generated from repeated readings of the transcripts and subsequently organized into categories and overarching themes. However, coding was undertaken by a single trained coder, and intercoder reliability was not formally assessed. Although the coding process followed established qualitative research methods and was reviewed for internal consistency by members of the research team, the absence of formal intercoder reliability testing may have introduced subjectivity in theme interpretation. Finally, GP trainees’ inability to follow-up with the same patients owing to rotational schedules reduced the opportunity to apply MI processes comprehensively.

### Implications of Findings for Practice

MI may assist in addressing behavioral and relational barriers to diabetes care—such as patient ambivalence, low motivation, or resistance to change—we acknowledge that MI alone cannot resolve the full range of complex challenges associated with chronic disease management. Structural issues, resource constraints, and system-level determinants also contribute significantly to outcomes. Thus, MI should be viewed as a single component of a broader multifaceted strategy aimed at enhancing communication, empowering patients, and supporting behavioral change.

While MI is often perceived as time-consuming, the use of brief MI, as emphasized in our training, can actually help structure consultations more efficiently. Several participants reported that once familiar with brief MI, they were able to guide patient discussions more purposefully, avoid redundant advice-giving, and promote greater patient involvement in setting goals. Over time, this may reduce the burden on clinicians by improving communication flow and minimizing repeated discussions of unresolved issues in future visits. Thus, brief MI may mitigate, rather than exacerbate, the challenges posed by time-limited practice environments. The development of brief MI protocols tailored to high-volume clinical settings could enhance feasibility without compromising effectiveness. MI’s emphasis on patient autonomy and behavioral change aligns with modern health care priorities, making it a valuable tool in chronic disease management. To support GPs in integrating MI into their daily practices, it is essential to provide continued education, peer coaching, and expert supervision. Policymakers should consider supporting MI training as part of CPD programs, given its demonstrated benefits in improving patient outcomes.

Further studies should explore the key components of brief MI that can retain the effectiveness of full-process MI while remaining practical in busy clinical environments. In addition, future studies should include longer follow-up periods to assess the sustained impact of MI on both GPs’ skills retention and patient outcomes over time. Research should also focus on optimizing MI training approaches by identifying effective educational methods suited to GPs, as well as strategies to increase uptake and sustained implementation of MI in routine practice. The potential of digital tools, such as mobile apps or web-based coaching platforms, to support MI training and delivery warrants further exploration to enhance accessibility and long-term integration into primary care.

### Conclusions

This study underscores the transformative potential of MI in primary care, particularly in diabetes management. MI training not only enhanced GP trainees’ knowledge, but also translated into tangible improvements in patient health metrics. Brief MI is an alternative if one cannot perform a full session of MI and better suits the busy schedule of GPs. However, challenges such as time demands, skill mastery, and patient continuity must be addressed to maximize its usability. By refining training methods, supporting GPs in their implementation efforts, and exploring innovative applications of MI, health care systems can better harness its benefits to improve patient outcomes and advance the quality of care.

## Supplementary material

10.2196/75916 Multimedia Appendix 1 Brief motivational interviewing guides.

## Abbreviations

CME: continuing medical education
CPD: continuing professional development
GP: general practitioner
MI: motivational interviewing
