# Supplementary material for: Impact of Motivational Interviewing Education on General Practitioners’ and Trainees’ Learning and Diabetes Outcomes in Primary Care: Mixed Methods Study
Source: JMIR Med Educ. 2025 Sep 16;11:e75916. doi: 10.2196/75916 (PMC12443351; doi:10.2196/75916)
Supplement: Multimedia Appendix 1 [file mededu-v11-e75916-s001.docx]

**Brief Motivational Interviewing (MI) Guides**

**Note:** Before using this brief MI guide, physicians may begin by applying the fundamental skills (OARS) to engage the patient.

| **Physician/Counselor** | **Patient** |
| --- | --- |
| 1. Since being diagnosed with diabetes, if you were to rate yourself on managing your diet and/or exercise on a scale of 1 to 10, with 10 being the best, what score would you give yourself? |  |
| 2. Why didn’t you give yourself a full score? |  |
| 3. Regarding your diet and dietary control, do you think there are any changes you’d like to make in the next month? (**Focusing**)  And why would you want to make this change? |  |
| 4. If you decide to change/control your diet, how might you go about it to make it successful? (**Evoking**: open-ended question: D**A**RN) |  |
| 5. Please give the three best reasons of reducing sweet foods/dietary control. (**Evoking**: open-ended question: DA**R**N) |  |
| 6. On a scale of zero to ten, how important is it for you to reduce sweet foods/control your diet? (0 = not important at all, 10 = the most important thing in your life right now) What number would you say? (**Evoking**: important ruler) |  |
| 7. Why did you choose …….. (that number) and not zero? |  |
| 8.You rated the importance of this change as ……(the selected number), which means that you see importance in changing your diet control because ….……….(the three best reasons that patient provided) (**Responding to change talk**: EA**R** (simple reflection) S) | |
| 9. We’ve been discussing dietary changes for a while now. Let me summarize what we’ve talked about. (**Responding to change talk**: EAR**S**)  -The change you want to make regarding your diet is …………………………………………...................................  -You rated the importance of reducing sweet foods/diet control at ……………………………………………………  -It is importnace because (reasons that patient gave for question no.7) ………………………………………………  -if you succeed, it will (three best reasons that patient provided) ……………………………………………………… | |
| 10. So, what do you think you will do? (**Planning**) |  |
| **Stage of Change (Before Counseling)** | **Stage of Change (After Counseling)** |
| □ Pre-contemplation  □ Contemplation  □ Determination  □ Action  □ Maintenance  □ Relapse | □ Pre-contemplation  □ Contemplation  □ Determination  □ Action  □ Maintenance  □ Relapse |
